# Wheelchair skill tests in wheelchair Basketball: A systematic review

**DOI:** 10.1371/journal.pone.0276946

**Published:** 2022-12-01

**Authors:** Carlos Mariano Aguiar Ferreira da Silva, Karina Santos Guedes de Sá, Andreia Bauermann, Mariane Borges, Minerva de Castro Amorim, Mateus Rossato, José Irineu Gorla, Anselmo de Athayde Costa e Silva

**Affiliations:** 1 Graduate Program in Neurosciences and Behavior, Federal University of Pará, Belém, Brazil; 2 Laboratory of Adapted Physical Activity, Federal University of Pará, Belém, Brazil; 3 Faculty of Physical Education, University of Campinas, Campinas, Brazil; 4 Researcher of the Brazilian Paralympic Academy, Brazilian Paralympic Committee, São Paulo, Brazil; 5 Graduate Program in Human Movement Sciences, Federal University of Pará, Belém, Brazil; 6 Faculty of Physical Education, Federal University of Amazonas, Manaus, Brazil; Sport Sciences School of Rio Maior - Politechnic Institute of Santarem, PORTUGAL

## Abstract

**Objectives:**

The aim of this study was to identify, describe and synthesize the skill tests used in wheelchair basketball.

**Method:**

A systematic review was carried out in the databases: PubMed/Medline, ScienceDirect, Scopus, Web of science and Google Scholar from inception to January 2021 with up to date in January 2022. the eligibility criteria used were Inclusion: (i) evaluation of wheelchair basketball athletes; (ii) using skill tests (defined as agility, speed, ball maneuverability, slalom, etc.) and (iii) papers needed to be written in English and published in peer-reviewed journals. Exclusion: (i) papers with poor description of the test methodology, (ii) participants not classified as wheelchair basketball athletes (less than one year of practice) and (iii) Participants were not people without disabilities.

**Results:**

Our main findings were: a) the most explored skills were pass and speed, and the most frequent test was the pass tests and sprint tests, b) Strong associations were found between sports classes and performance in field tests, c) The most used tests for each skill were: pass = pass accuracy and maximum pass; speed = 20m sprint test with and without the ball; agility = slalom test; dribbling = obstacle dribbling tests and throw = free throw and spot shot.

**Conclusion:**

The most explored skills were passing and speed, and to evaluate these skills we highlight the two-handed chest pass test, 20m sprint test with ball and the WMP test. The use of specific tests can facilitate the creation of reference standards and possible comparison of athletes and, thus, enable better training conditions, aiming to meet the specific demands of each athlete and team.

## Introduction

Wheelchair basketball is one of the most popular paralympic sports, with growing popularity and international competitions being held around the world. The growing level of professionalism and growing interest demand a more scientific view of the sport. Wheelchair basketball is played by two teams made up of individuals with compromised lower limbs, by amputation, paralysis, etc. As it is a high intensity intermittent/interval sport [1] it is important that athletes have physical skills such as speed, agility, strength, power, endurance and technical skills such as pushing, turning, kicking, hitting, dribbling, throwing, passing and catching the ball [2], as this is an intermittent modality [1].

Field-specific tests used in wheelchair basketball comprise actions and skills of the game. The execution of technical standards and development of dynamism of the game constitute a complex structure of skills influenced by factors such as sensorimotor, intellectual, social interaction skills, coordination, and team skills [3]. Laboratory tests do not seem to be sufficient for coaches in their assessments who prefer adapted field tests composed by sport-specific skills, propulsion techniques and individual adaptations [4].

In this context, the use of skill tests is interesting both for the assessment of these skills to determine training strategies to improve performance, as well as for tactical decision during competitive matches. The importance of skill assessment in wheelchair basketball has grown in recent years and we believe it is fundamental to the development of the sport. However, to the best of our knowledge, we have not found studies that have summarized the evidence regarding to the main court tests used to assess wheelchair basketball skills. Therefore, the objective of this study was to identify, describe, and synthesize the skill tests used in wheelchair basketball to delimit the state of the art of this topic, which is useful for those interested in using skills tests and also allows for creating bases for the proposal of test batteries.

## Methods

This systematic review is presented in accordance with the statement Preferred Reporting Items for Systematic Reviews and Meta-Analysis (PRISMA) [5], (please, see S2 Table). The systematic review protocol was registered with the International Prospective Register of Systematic Review (PROSPERO; available at: https://www.crd.york.ac.uk/PROSPERO/) on 28 May 2020 (registration number: CRD42020159566) [6]. The research question and other systemic review procedures were addressed with reference to the following PICO strategy: Participants (wheelchair basketball athletes); Intervention (Assessment of wheelchair skills); Comparison (validation of skill tests and/or assessment of skill tests); Outcomes (main batteries of test, locals where tests were applied, history of skill tests, how many athletes were assessed, reference scores). Following this PICO strategy, our research question was as follows: What are the skill tests used in wheelchair basketball assessments?

### Eligibility criteria

The systematic search comprised observational studies written in English, prospective or retrospective and cohort studies. Studies were eligible for inclusion according to the following criteria: (i) evaluation of wheelchair basketball athletes; (ii) using skill tests (defined as agility, speed, ball maneuverability, slalom, etc.) and (iii) papers needed to be published in peer-reviewed journals. Studies were excluded from the analysis based on the following criteria: (i) articles with poor description of the test methodology, (ii) participants not classified as wheelchair basketball athletes (less than one year of practice) and (iii) Participants were not people without disability.

### Search strategy

Searches of the electronic databases PubMed/Medline, ScienceDirect, Scopus, Web of Science, and Google Scholar were performed from inception to January 2021 with up to date in January 2022. Articles were retrieved from electronic databases using the following search strategy: "wheelchair basketball" AND performance OR "Athletic Performances" OR "Sports Performance" OR "Functional Performance" OR "Physical Performance" AND "field tests" OR "skill tests". These words needed to occur in the title or in the abstract of the manuscript.

### Selection process and data collection process

**Fig 1:**
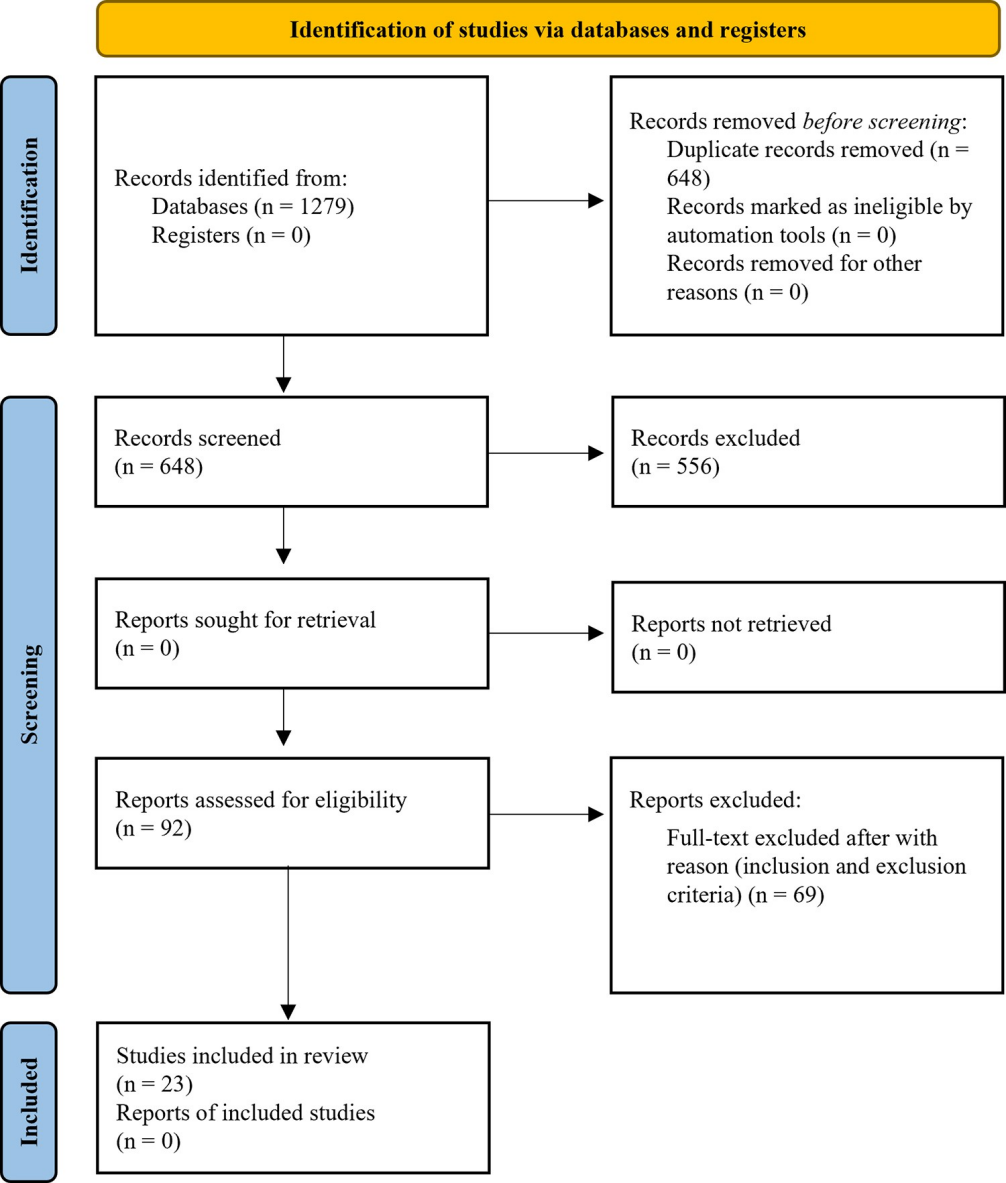
PRISMA flow diagram of the studies’ selection. Figure based on https://prisma-statement.org/prismastatement/flowdiagram.aspx [5].

Identified articles in systematic search were initially checked for relevance by two independent researchers (CMAFS and KSGS). The articles were selected after a sequential reading of the title and abstract, always in this order. Subsequently, the researchers reviewed the full texts of potentially eligible articles. A third researcher (AACS) resolved any disagreement regarding the inclusion of the study among reviewers. Fig 1 shows the flow chart of the studies during the study selection process. The records retrieved from databases are available in S1 Table.

Data collection was performed by two independent researchers (CMAFS and KSGS), supported by a third researcher (AACS) when necessary. Data extracted from studies included objectives, sample (number, sport class, and training level), skill tests used, main outcomes, and results of the skill tests.

### Study risk of bias assessment

The quality of the study was evaluated by two researchers (CMAFS and KSGS) according to the Evaluation Tool for Cross Sectional Studies (AXIS tool) [7], which is considered an appropriate tool to assess the methodological quality of studies in systematic reviews. This tool consists of twenty questions divided in five groups (introduction, methods, results, discussion and other) that evaluate the quality and risk of bias of cross-sectional studies.

## Results

### Study selection

Through searches, a total of 1279 articles were found (Fig 1 and Table 1). These studies were inserted into an excel sheet (© Microsoft Corporation) and duplicates were removed, leaving 648 studies. Titles and abstracts were read and 92 manuscripts were selected; after that, inclusion and exclusion criteria were applied, and for these review 23 articles were remaining.

**Table 1:** Search strategy.

	Articles Found				
	PubMed	Scopus	Science Direct	Google Scholar	Web of Science
"wheelchair basketball" AND performance OR "Athletic Performances" OR "Sports Performance" OR "Functional Performance" OR "Physical Performance" AND "field tests" OR "skill tests"	175	*	*	372	*
"wheelchair basketball" AND "field tests" AND performance	14	12	15	312	10
"wheelchair basketball" AND "field tests" AND "Athletic Performances"	0	0	3	1	0
"wheelchair basketball" AND "field tests" AND "Sports Performance"	0	1	3	22	0
"wheelchair basketball" AND "field tests" AND "Functional Performance"	0	0	0	32	0
"wheelchair basketball" AND "field tests" AND "Physical Performance"	2	5	0	107	2
"wheelchair basketball" AND "skill tests" AND performance	1	1	7	139	1
"wheelchair basketball" AND "skill tests" AND "Athletic Performances"	0	0	1	2	0
"wheelchair basketball" AND "skill tests" AND "Sports Performance"	0	0	1	2	0
"wheelchair basketball" AND "skill tests" AND "Functional Performance"	0	0	1	6	0
"wheelchair basketball" AND "skill tests" AND "Physical Performance"	0	0	2	35	0
**Total without duplicates**	**180**	**13**	**23**	**421**	**11**

Subtitle: (*) disregarded search due to the large number of articles.

### Study participants

A total of 838 athletes competing at different playing standards. Of the 23 studies, 17 divided their athletes by sport class (class 1.0–1.5 = 182 athletes, class 2.0–2.5 = 196 athletes; class 3.0–3.5 = 164 athletes, and class 4.0–4.5 = 124 athletes). Three studies divided it into category A (Functional Classification Class 1.0–2.5) and B (Functional Classification Class 3.0–4.5) [8–10]. One study divided into an intervention group and a control group [11]. One study divided into elite wheelchair basketball players at different league levels [12]. A study divided according to medical classification [13].

### Study description

In Table 2 are described the data extraction. Regarding applied skill tests, we find that pass tests (speed pass, pass accuracy, pass for distance, two-handed chest pass, medicine ball pass, maximal pass) were used in 11 articles [2,3,12,14–21]. Sprint tests (20 m sprint test without and with ball, 5 m sprint test without and with ball, 3 m sprint test, 10 m sprint test, 10x5 m sprint test, 30 seconds sprint test) were used in 13 articles [2,12,16–18,20,22–28].

**Table 2:** Data extraction.

Reference	Objectives	Sample	Skill tests	Main Results	Results of the skill tests
**Brasile (1986) [14]**	Evaluate the relationship between the skills proficiency levels and the NWBA classification level of athlete.	Were selected 91 male players (high performance nation level) from 18 NWBA teams. NWBA classification: class 1 = 26 class 2 = 34 class 3 = 31	8 tests were applied: - 20m push (speed). - free-throw shooting (throw). - obstacle dribble (ball hand speed and agility). - basket per minute (shooting). - rebounding (rebound and catching). - speed pass (pass and catching). - pass for accuracy (passing).	The findings indicated non-significant differences in scores across disability NBWA classification levels in six of the test items scores. Pass for accuracy test presented significant difference between classes (p<0.01).	- Free throws (score): class 1 = 6.19 class 2 = 6.97 class 3 = 7.22 - Speed pass (score): class 1 = 12.61 class 2 = 12.41 class 3 = 13.61 - Rebound (score): class 1 = 14 class 2 = 15.38 class 3 = 16.87 - One-minute shooting (score): class 1 = 6.15 class 2 = 7.50 class 3 = 8.35 - Pass for accuracy (score): class 1 = 21 class 2 = 13.38 class 3 = 15 - 20m sprint (s): class 1 = 6.87 class 2 = 6.76 class 3 = 6.55 - Obstacle dribble (s): class 1 = 54 class 2 = 50 class 3 = 49
**Brasile (1990) [24]**	Investigation of the influence of specific NWBA classification level, along with additional independent variables that may also influence the performance levels of wheelchair basketball participants.	79 male players were selected from NWBA/Paralyzed Veterans of America (PVA) wheelchair basketball (National, High Performance): class 1 = 25 class 2 = 37 class 3 = 17	5 tests were applied: - pass for accuracy. - 20m sprint (speed). - obstacle dribble (ball hand, speed and agility). - minute shot (d) (shooting). / minute shot (nd) (shooting). - and spot shot (shooting).	The results indicate that the participant’s NWBA player classification level is the most important predictor of the total overall skill score. Individual univariate tests showed a significant difference across disability levels for two of the seven items, the 20-meter sprint and the minute shot non-dominant hand.	- Pass for accuracy(D-score): class 1 = 16.2 class 2 = 18.4 class 3 = 19.2 - Pass for accuracy (ND-score): class 1 = 6.5 class 2 = 8.8 class 3 = 10 - 20m sprint (s): class1 = 7.1 class2 = 6.6 class 3 = 6.5 - Obstacle dribble (s): class1 = 45.8 class 2 = 43.7 class3 = 43 - One minute shot (D score): class 1 = 6.7 class 2 = 9.7 class 3 = 9.7 - One-minute shot (ND-score): class 1 = 3.2 class 2 = 4 class 3 = 5.5 - Spot shot (score): class 1 = 17.8 class 2 = 18.2 class 3 = 18.4
**Vanlandewijck et al.(1999) [15]**	Develop a field test battery which could be used by trainers/coaches to evaluate the players’ overall wheelchair basketball performance. In this study ‘overall’ wheelchair basketball performance includes the aerobic and anaerobic capacity and specific wheelchair basketball skills.	Were selected 46 male wheelchair basketball players representing eight Belgian teams, all with at least 2 years of experience in the Belgian National League. Medical examination identified: -5 athletes with spina bifida, -5 with spastic diplegia, -7 with a complete spinal cord injury (lesion level thoracic 3 to lumbar 1), -6 with an incomplete spinal cord injury (lesion level thoracic 3 to lumbar 4), -4 with polio, -12 with an amputation. - Seven experienced able-bodied players completed the sample. -Functional classification system (International Wheelchair Basketball Federation: -1 and 1.5 point [n = 13], -2 and 2.5 point [n = 9], -3 and 3.5 point [n = 6], -4 and 4.5 point [n = 11]).	Six tests were applied: -20 m sprint test. - Lay-up test. - Figure-eight + ball test. - Zone-shot test. - Figure-eight test. - Pass for accuracy test.	General field testing results Mean test duration per wheelchair basketball team for the whole test battery was 1 h 22 min ± 10.18 min. Scoring accuracy of the players was very high, ranging from 95 to 100%. The lowest scoring accuracy was observed during the pass for accuracy test. Frequent mistakes were faulty interpretations of wheelchair dribbling violations.	-20m sprint test (s): Test: 5.93 (0.21) Retest: 6.22 (0.14) -Lay-up test: Test: 27.48(6.82) Retest: 28.26(7.66) -Figure eight + ball: Test: 13.67(1.08) Retest: 13.67(1.14) -Figure eight Test: 17.33(1.21) Retest: 17(1.09) -Zone-shot: Test: 28.38(7.66) Retest: 27.29(8.61) -Pass accuracy: Test: 31.83(9.54) Retest: 31.33(7.79)
**Zwakhoven et al. (2003) [19]**	Develop an observation protocol in which seven specific ball-handling skills (dribble, bounce-stop, bounce-spin, passing, catching, shot and lay-up) were described in a highly mature way.	Were selected 48 male players (high performance national level) divided two phases. 1° = 16 best world players (two per class). 2° = 48 players subdivided: - elite = the same sixteen players used in the first phase of this study. - sub-elite = 16 - advanced = 16	Six tests were applied: - Dribble (dribble). - bounce stop (dribble and control). - bounce-spin (dribble and control). - passing (pass). - Catching (accuracy). - shot and lay-up (agility).	Test-retest correlations were high for all parameters (.76 to 1.00), except for dribble (.62). Correlations between subscores were moderate (.53 to .61). The correlations between the subscores and the total score were high (.74 to .89). Shows no significant differences between elite and sub-elite players on all scores. Scores are slightly higher in the elite group. Advanced players scored significantly lower in SS2, SS3, and TOT compared to the elite and sub-elite group. There were no significant differences between the IWBF classes and NWBA classes on all subscores and the total score.	Score percentages: - Elite: SS1 = 68.6 SS2 = 87.2 SS3 = 59.6 - Subelite: SS1 = 63.6 SS2 = 83 SS3 = 54.7 - Advanced: SS1 = 52.3 SS2 = 71.3 SS3 = 40.5 IWBF classification: - class 1: SS1 = 53.1 SS2 = 77.6 SS3 = 43.2 - class 2: SS1 = 63.4 SS2 = 80.6 SS3 = 49.1 - class 3: SS1 = 61.7 SS2 = 80.1 SS3 = 50.2 - class 4: SS1 = 63.7 SS2 = 81.5 SS3 = 58.9 NWBA classification: class 1: SS1 = 53.1 SS2 = 77.6 SS3 = 43.2 class 2: SS1 = 62.5 SS2 = 80.9 SS3 = 49.8 class3: SS1 = 63.5 SS2 = 80.9 SS3 = 56.1
**Doyle et al. (2004) [25]**	Determine whether the performance of the wheelchair athlete could further support a reduction in the classification classes for wheelchair basketball.	46 male players were selected from NWBA summer basketball camps held in two consecutive years. (National Intercollegiate Division).	Teste applied: - 20m sprint (speed).	Differences between groups for both time and velocity over the entire 20-meter distance. (p<0.05).	Over the entire distance of 20 meters, Class 1 players (lower functioning) were slower than Class 2 and 3. Statistically, all groups performed equally for both time and velocity over the shorter 1 and 5 meter intervals.
**Harbalis et al. (2008) [11]**	Examine the effectiveness of a self-talk intervention program on the performance of wheelchair basketball players in two fundamental skills, dribbling and passing.	22 male players were selected from two different clubs in the same league (national/high performance). - intervention group = 10 - control group = 12	2 tests were applied: dribbling test (dribble). Pass the test (pass).	Overall, that dribbling and passing performance for the experimental team improved more than that of the control team.	Descriptive statistics (mean). Intervention group: - dribbling performance(sec): pre = 23.84 mid = 21.65 post = 20.80 - passing performance(score): pre = 9.10 mid = 15.10 post = 15.20 Control group: - dribbling performance(sec): pre = 18.85 mid = 18.71 post = 18.57 - passing performance(score): pre = 15.50 mid = 15.58 post = 16.33
**Yanci et al. (2015) [28]**	To determine the reliability and reproducibility of an agility T-test and Yo-Yo 10 m recovery test and to analyse the physical characteristics measured by sprint, agility, strength and endurance field tests in wheelchair basketball (WB) players.	16 wheelchair basketball players, 14 males and 2 females, belonging to the Spanish national WB third division league. -6 athletes with Spinal cord injury. -2 with amputation. -1 with spinal cord injury + amputation. -1 with Viral disease (polio). -1 with Dermoid cyst (embryonic origin). -1 with Legg-Calve-Perthes. -1 with Dysplasia. -2 spina bifida. -1 with Cauda equina syndrome.	Six tests were applied: -Sprint Without and with ball. -Maximal sprint test with the ball. -T-test. -Pick-up the ball. -Maximal pass. -Yo-Yo intermittent recovery test.	The main contribution of the present study is the characterization of the physical performance profile of WB players using a field test battery. Consequently they may be appropriate instruments for measuring physical fitness in WB.	-T-test (s). Test: 16.96 (1.14) Retest: 16.85(1.20) -Yo-Yo intermittent recovery test (m). Test: 1014(369). Retest: 991(358). -Pick-up the ball (s): 16.05(2.52) -Maximal pass (m): 8.39(1.7)
**Cavedon et al.** **(2015) [20]**	Assess anthropometry, body composition, and performance in sport-specific field tests in a national sample of young Italian wheelchair basketball players, as well as assess the association of these variables with the players’ functional ability rating and game-related statistics.	52 wheelchair basketball players,45 male and 7 female. Participating in the 2013–2014 season Of Italian Young Wheelchair Basketball Championship. Disabilities comprised spinal cord injury (incomplete tetraplegia, n = 1; complete/incomplete paraplegia, injury level C6-T12, n = 6), other comparable neurologic disorders (n = 5), spina bifida (n = 17), cerebral palsy (n = 19), phocomelia (n = 2), lower extremity poliomyelitis (n = 2). Functional classification -0.5 points, n = 19 -1.0 points, n = 8 -1.5 points, n = 8 -2.0 points, n = 3 -2.5 points, n = 5 -3.0 points, n = 5 -3.5 points, n = 2 -4.0 points, n = 2 –The single 4.5 points.	Seven tests were applied: -5m sprint -20m sprint with ball -Suicide -Maximal pass -Pass for accuracy -Spot shot -lay-ups	The main contribution of the age, WB experience and FM% do not influence WB performance. Sitting height positively contributes to WB performance. Maximal pass, lay-ups and upper arm circumference, alone or in combination with each other, significantly predict game point scoring and should be carefully considered in young WB physical and technical training plans. Large overlapping in both sportspecific field tests performance and game-related statistics is present in younger WB players belonging to functional ability Class B, C, and D.	Performance in sport-specific field tests: -5m sprint (s): 2,7(0.66) -20m sprint with ball (s): 9.2(3.07) -Suicide (s) 51.2(12.17) -Maximal pass (n): 7.8(3.40) -Pass for accuracy (n); 15.9(7.85) -Spot shot (n) 19.4(11.83) -lay-ups (n): 10.7(6.91)
**Cavedon et al.** **(2018) [21]**	To investigating anthropometry, body composition, and performance in sport-specific field tests of female WB players as well as exploring sex-related differences.	42 wheelchair basketball players, 26 male and 16 female. the various Italian Wheelchair Basketball Championships (A1 League, A2 League, B League and Young). The disabilities of the female WB players included spinal cord injury (complete/incomplete paraplegia, n = 6), spina bifida (n = 2), phocomelia (n = 1), lower extremity poliomyelitis (n = 2), spastic tetraparesis (n = 1), and unilateral above-knee amputation (n = 1). The disabilities of male WB players comprised spinal cord injury (complete/incomplete paraplegia, n = 5), spina bifida (n = 7), lower extremity poliomyelitis (n = 4), spastic tetraparesis (n = 4), spastic paraparesis (n = 1), spastic diplegia (n = 1), and cerebral palsy (n = 4). The median Point value (interquartile range) was 2.0 (1.75) and 2.0 (1.72) in the female and male groups, respectively.	10 tests were applied: - Pass for accuracy (pass). - 5m sprint (speed). - free-throw shooting (shot). - 20m Sprint with ball (speed and control). Maximal pass (pass and accuracy). - slalom (agility). - lay-ups (accuracy). - pick up the ball (control). - spot shot (shot). - suicide (resistance).	Female and male WB players differ in several upper body anthropometric variables and females show greater %FM. Sex-related differences exist in the performance of male and female WB players, which are mainly associated with passing (explosiveness) and resistance ability. Sex-related differences in performance might be fully compensated if 1.5 functional points are subtracted from female WB players.	Sport-specific field test: Subgrupo A -5m sprint (s): Female:2.1(0.3) Male: 2.4(0.6) -20m sprint with ball (s): Female: 7.8(1.3) Male: 7.3(0.9) -suicide (s): Female: 54.2(7) Male: 45.9(8.) -Lay-ups (n): Female: 15.8(8.9) Male: 13.9(5.1) -Pass of accuracy (n): Female: 16.7(8.7) Male 16.9(7.5) -Maximal pass (m): Female: 8.1(1.7) Male: 9.6(2.8) -Spot-shot (n): Female: 24.2(13) Male: 21.8(6) Subgrupo B -5m sprint (s): Female:2.2(0.4) Male: 2.6(0.5) -20m sprint with ball (s): Female: 7.6(1.2) Male: 8.1(1.5) -suicide (s): Female: 53.9(6.7) Male: 47.7(8) -Lay-ups (n): Female: 16.3(8.9) Male: 10.9(6.1) -Pass of accuracy (n): Female: 16.2(8.4) Male 16(8.5) -Maximal pass (m): Female: 8.2(1.7) Male: 8.5(1.7) -Spot-shot (n): Female: 25.3(13.7) Male: 24.1(7.7)
**Yüksel and Sevindi. (2018) [12]**	Examine the anthropometric and biometric features of the elite wheelchair basketball players in different league levels, and evaluate with regard to field tests particular to wheelchair basketball.	21 male players were selected from 68 Aksaray Municipality Sports Club in Turkey Wheelchair Basketball First League and Second League (high performance national / international). - congenital = 3 - acquired disabled = 18	Eight tests were applied: - pass for distance(pass). - 20m speed(speed). - slalom without the ball(agility). - slalom with the ball(agility and control). - lay up(agility and control). - zone shot(shooting). - pass for accuracy(pass). - 6min endurance race(resistance).	The significant difference between the average values of the First and Second League wheelchair players, it was determined that there was a statistically significant difference (p < 0.05) on the pass for distance (p = 0.043), 20-m speed (p = 0.043), slalom without the ball (p = 0.001), slalom with the ball (p = 0.013), zone shot (p = 0.014), pass for accuracy (p = 0.020), and 6-min endurance race test (p = 0.007) parameters, while statistically there was no significant difference (p > 0.05) on the other parameters.	Measurements: - pass for distance(m): group1: min = 8.40 max = 12.20 mean = 10.65 group2: min = 6 max = 12.50 mean = 9.04 - 20m speed(s): group1: min = 5.16 max = 6.60 mean = 5.75 group2: min = 5.45 max = 6.90 mean = 6.20 - slalom without the ball(s) group1: min = 11.03 max = 13.69 mean = 12.15 group2: min = 12.90 max = 16.80 mean = 13.91 - slalom with the ball(s) Group 1: min = 12.50 max = 17.02 mean = 14.28 Group 2: min = 14.76 max = 25 mean = 16.89 - lay up(score) group 1: min = 19 max = 26 mean = 23.58 group 2: min = 14 max = 29 mean = 22.77 - zone shot(score) group 1: min = 23 max = 38 mean = 32.08 Group 2: min = 22 max = 34 mean = 26.55 - pass for accuracy(score) Group 1: min = 10 max = 32 mean = 26 group2: min = 3 max = 30 mean = 19.66 - 6min endurance race(m) group1: min = 1032 max = 1311 mean = 1203.91 group2: min = 790 max = 1140 mean = 1140
**Skucas et al. (2009) [3]**	State and evaluate the playing skills of wheelchair basketball players in different game positions (a playmaker, a forward, a center) in official competitions.	32 male players with national and international level (high perfomance) divided into three groups that participated according to their position during the game: - playmakers = 9 - forwards = 11 - center = 12	5 tests were applied: - playing time (time). - passing (pass). - dribbling (dribble). - shooting (shot). - free shots accuracy (shot and control).	Quantitative and qualitative playing results and the results of integral preparation of wheelchair basketball players center position were significantly better than those of the playmakers and forwards.	Playmakers: - time played(min): mean = 32 the best = 40 - passing (score): mean = 13 the best = 23 - dribbling(score): mean = 9 the best = 25 - shooting accuracy (%): mean = 30 the best = 33 - free shots accuracy (%): mean = 25 the best = 0 forwards - time played(min): mean = 33 the best = 37 - passing(score): mean = 13) the best = 23 - dribbling(score): mean = 10 the best = 19 - shooting accuracy (%) mean = 30 the best = 42 - free shots accuracy (%) mean = 33% the best = 42. Center forwards - time played(min) mean = 33 the best = 40 - passing mean = 20 the best = 28 - dribbling mean = 20 the best = 29 - shooting accuracy (%): mean = 3 the best = 39 - free shots accuracy (%): mean = 40 the best = 0
**Molik et al. (2010) [17]**	Evaluate wheelchair basketball skills in athletes representing the different levels of functional classification and various types of disabilities.	Were selected 109 male wheelchair basketball athletes NWBA (national high perfomance). class 1 = 26 class 2 = 25 class 3 = 24 class 4 = 16 class 4.5 = 18	Six tests were applied: - 20-m sprint (speed). - two-handed chest pass (pass). - slalom without the ball (agility and control). - slalom with the ball (agility and control). - modified Cooper 12-minute test (resistance). - envelope drill.	The results demonstrated that there were observable differences between the skill test performance and the functional classes; the higher functioning classes performed better. However, there were no significant differences between functional classes 1 and 2, as well as between classes 3 through 4.5.	- sprint 20m(s): class 1 = 6.54 class 2 = 6.24 class 3 = 5.68 class 4 = 5.49 class 4.5 = 5.63 - two handed chest pass(m): class 1 = 8.89 class 2 = 9.52 class 3 = 10.49 class 4 = 11.81 class 4.5 = 11.09 - slalom without the ball(s): class 1 = 10.98 class 2 = 10.18 class 3 = 9.22 class 4 = 9.05 class4.5 = 9.92 - slalom with the ball(s): class1 = 14.40 class 2 = 12.79 class 3 = 10.92 class 4 = 10.47 class 4.5 = 11.93 - modified cooper test(m): Class 1 = 1748 class 2 = 1803 class 3 = 1971 class 4 = 2070 class 4.5 = 2002 - envelop drill(s): Class 1 = 26.22 class 2 = 26.19 class 3 = 23.18 class 4 = 22.69 class4.5 = 24.94
**De Groot et al. (2012) [22]**	Investigate the reliability and validity of wheelchair basketball field tests	We selected 19 wheelchair basketball players (high national proficiency). class 1.5 = 2 class 2 = 2 class 2.5 = 3 class 3.5 = 1 class 4 = 5 class 4.5 = 6	10 tests were applied: - Pass for accuracy (pass). - 5m sprint (speed). - free-throw shooting (shot). - 20m Sprint with ball (speed and control). -Maximal pass (pass and accuracy). - slalom (agility). - lay-ups (accuracy). - pick up the ball (control). - spot shot (shot). - suicide (resistance).	The results suggest that wheelchair basketball field tests are reliable and valid, except for shooting and passing items, which should be interpreted carefully.	Competition level Premier League: pass for accuracy points = 22.4 5m sprint = 2.4s free throws points = 18.4 20m sprint of the ball = 7s maximal pass = 14.6m slalom = 13.6s lays up points = 20.7 pick up ball = 15.3 s spot shot points = 37.4 suicide = 47.8s Tournament A: pass for accuracy points = 19 5m sprint = 2.5s free throw points = 12.5 20m sprint the ball = 7.4s maximal pass = 13.4m slalom = 13.6s lays up points = 17.4 pick up ball = 16.1s spot shot points = 30.6 suicide = 52 s Tournament B pass for accuracy points = 16.7 5m sprint = 2.6s free throws points = 15.6 20 m sprint of the ball = 8.7s maximal pass = 12.1m slalom = 16.1 s lays ups points = 12.5 pick up ball = 19s spot shot points = 25.9 suicide = 59.3s IWBF classification level Low (<4) pass for accuracy points = 20.4 5m sprint = 2.5s free throws points = 15.9 20m Sprint the ball = 7.8s maximal pass = 12m slalom = 14.6 s lays up points = 17 pick-up ball = 16.7s spot shot points = 31.6 suicide = 52.7s High (>4) Pass for accuracy points = 18.7 5m sprint = 2.5s free throws points = 15.8 20m Sprint the ball = 7.7s maximal pass = 14.4 m slalom = 14.5s lays ups points = 17.1 pick up ball = 17s spot shot points = 31.2 suicide = 53.8
**Molik et al. (2018) [8]**	to evaluate the associations between anaerobic performance, applicable field tests, and the functional classification levels in female wheelchair basketball athletes.	23 Female wheelchair basketball athletes (Category A, n = 9; six paraplegics, one tetraplegic, one with spina bifida, and one had cerebral palsy). Category B, n = 14; two with spina bifida, one had cerebral palsy, four with lower limb amputations, five had a minimal disability, and two had paresis of a lower limb) from the Canadian national team.	The tests applied was: -chest pass -shooting -5 m sprint -20 m sprint -slalom (with the ball) -slalom (without the ball)	Found that there were significant differences between the level of AnP and the employed field tests. Demonstrated that there were correlations between the functional classification of wheelchair basketball players and the achieved field test results and the analyzed parameters of AnP.	Results obtained: Category A: Chest pass (m): 8(1.1) Shooting(%): 40.7(24.3) 5m sprint(s): 2.2(0.1) 20m sprint(s): 6.4(0.3) Slalom without ball(s): 10.5(0.9) Slalom with ball(s): 12.4(2.6) Category B Chest pass (m): 9.8(1.7) Shooting(%): 53.4(19.6) 5m sprint(s): 2(0.2) 20m sprint(s): 5.9(0.4) Slalom without ball(s): 9.5(0.6) Slalom with ball(s): 10.7(1.4)
**Bergamini et al. (2015) [23]**	(I) Proposal of a method to identify biomechanical performance indicators of wheelchair propulsion using an instrumented in-field test, and (II) development of a training program specifically for the considered population and evaluation of its efficacy using the proposed method.	12 athletes. 10 men and 2 women. *FCS* (functional classification score). FCS 1 = 2, FCS 2 = 4, FCS 2.5 = 2, FCS 3 = 3, FCS 4.5 = 2. Medical examination identified four athletes with paraplegia, three with myelomeningocele, two with poliomyelitis, one with spastic diplegia, one with a below-knee amputation, and one with a knee arthroprosthesis due to bone cancer. All participants were righthanded. Regional (junior wheelchair basketball). With at least two years of previous wheelchair basketball experience.	The test applied was: 20m sprint (speed)	For the EG, significant differences were obtained for Timing (Δ*t*), push cycle frequency (f), Progression Force (*Fp*), and Bilateral Symmetry (sym) when comparing those parameters before and after the administration of the training program. No significant difference was found between the two groups, for both second experimental session (ES2) and final experimental session (ES3).	Moderate correlations were found for all coefficients of variation (*CV*Δ*t*, *CVsym*, and *CVFp*), and a weak negative correlation was obtained for *Fp*. No significant differences were found between the second experimental session (ES2) and the final experimental session (ES3) for the CG. Conversely, for the EG, significant differences were obtained for Δ*t*, f, *Fp*, and sym when comparing those parameters before and after the administration of the training program. No significant difference was found between the two groups, for both ES2 and ES3.
**de Witte et al. (2017) [27]**	(1) to describe the development of a field-based wheelchair test that assesses mobility performance capacity and closely mimics the wheelchair mobility skills required in real wheelchair basketball matches, (2) to define the field-based test developed, and (3) to assess the construct validity and test-retest reliability of the newly developed field-based WMP test for wheelchair basketball.	validity study: 46 players; reliability study: 23 players/ - validity study: class 1–1.5 = 8, 2–2.5 = 11, 3–3.5 = 8, 4–4.5 = 19; Reliability study: class 1–1.5 = 2, 2–2.5 = 1, 3–3.5 = 5, 4–4.5 = 15 - validity study: competing at different playing standards; - reliability study: competing at a national playing standard	a single test with 15 activities was applied: WMP test (180° turn on the spot (left); 12 m sprint; 12 m rotation (right); 12 m rotation (left); 180° turn on the spot (right); 3-3-6 m sprint; 3-3-6 m rotation (left); 3-3-6 m rotation (right); 90°–90° turn on the spot with stop (left); 12 m dribble; 12 m rotation dribble (right); 12 m rotation dribble (left); 90°–90° turn on the spot with stop (right); Combination)	Males performed better than females (P < 0.001, effect size [ES] = −1.26) and international men performed better than national men (P < 0.001, ES = −1.62). Performance time of low (≤2.5) and high (≥3.0) classification players was borderline not significant with a moderate ES (P = 0.06, ES = 0.58). Reliability was excellent for the overall performance time (ICC = 0.95).	Overall performance time (sum activities): athletes with classification ≤2.5 = 79.25 (6.56); athletes with classification >2.5 = 75.95 (4.97)
**Tachibana et al. (2019) [18]**	To clarify field-based skill test items closely related to the functional classification level.	26 female players/ class 1 (classes 1.0 and 1.5) = 7; class 2 (classes 2.0 and 2.5) = 7; class 3 (classes 3.0 and 3.5) = 4; class 4 (classes 4.0 and 4.5) = 8 national	5 field-based skill tests: - 20 m sprint - Agility T-test - Figure-eight with a ball test - The Yo-Yo 10 m recovery test - Three types of maximal passes	Significant differences between classification levels were found for maximal one-handed passes (baseball and hook passes) and the figure eight with a ball test. Furthermore, performance in the 20 m sprint and 10 m Yo Yo recovery tests significantly differed between classes 1 and 4.	Class 1: 20 m sprint (s) = 6.25 ± 0.40; agility t-test (s) = 15.02 ± 1.08; figure eight with ball test (s) = 37.15 ± 5.79; Yo-Yo 10 m recovery test (m) = 657.14 ± 288.3; maximal chest pass (m) = 7.7 ± 1.1; baseball pass by dominant hand (m) = 7.6 ± 0.4 Class 2: 20 m sprint (s) = 5.82 ± 0.28; agility t test (s) = 14.12 ± 0.56; figure eight with ball test (s) = 31.07 ± 1.09; Yo-Yo 10 m recovery test (m) = 1042.9 ± 231.4; maximal pass chest pass maximal (m) = 8.6 ± 1.1; baseball pass by dominant hand (m) = 9.1 ± 1.3 Class 3: 20 m sprint (s) = 5.98 ± 0.32; agility t-test (s) = 14.26 ± 0.50; figure-eight with a ball test (s) = 33.00 ± 2.71; Yo-Yo 10 m recovery test (m) = 930.0 ± 429.1; maximal pass chest pass (m) = 8.8 ± 1.2; baseball pass by dominant hand (m) = 10.0 ± 2.1 Class 4: 20 m sprint (s) = 5.59 ± 0.33; agility t test (s) = 13.95 ± 0.90; figure eight with ball test (s) = 29.93 ± 1.14; Yo-Yo 10 m recovery test (m) = 1250.0 ± 240.5; maximal pass chest pass maximal (m) = 10.3 ± 1.5; baseball pass by dominant hand (m) = 12.3 ± 1.7
**Gil et al. 2015 [2]**	To ascertain if the IWBF classification, the type of injury and the wheelchair experience were related to different performance field-based tests.	13 players/ Class 1 = 1; class 1.5 = 1; class 2 = 3; class 2.5 = 1; class 3 = 2; class 3.5 = 2; class 4 = 2; class 4.5 = 1. National	Six tests were applied: - 5 and 20 m sprints; - 5 m and 20 m sprints with a ball; - T-test; - Pick-up test - modified 10 m Yo-Yo intermittent recovery test; - maximal pass - medicine ball throw	The IWBF class was correlated (p<0.05) to the hand dynamometry (r = 0.84), the maximal pass (r = 0.67) and the medicine ball throw (r = 0.67). While the years of dependence on the wheelchair were correlated with the velocity (p<0.01): 5 m (r = -0.80) and 20 m (r = -0.77) and agility tests (r = -0.77, p<0.01). Also, the 20 m sprint with a ball (r = 0.68) and the T-test (r = -0.57) correlated (p<0.05) with the experience of playing wheelchair basketball.	- Maximum pass (m) = 9.15 ± 1.72; - Medicine ball throw (m) = 3.78 ± 0.66; - 5 m sprint (s) = 1.86 ± 0.22; - 20 m sprint (s) = 5.65 ± 0.45; - 5 m sprint with a ball (s) = 2.09 ± 0.32; - 20 m sprint with a ball (s) = 6.56 ± 0.66; - T-test (s) = 16.94 ± 1.23; - Pick-up test (s) = 16.37 ± 2.69
**Marszałek et al. 2019 [16]**	To assess test-retest reliability of the newly developed field-based tests focused on short time efforts with maximal intensity for wheelchair basketball players.	9 male players/ Class 1 = 2; class 1.5 = 2; class 2 = 1; class 3 = 3; class 3.5 = 1. National	11 tests were applied: - 3 m sprint; - 5 m sprint; - 10 m sprint; - 20 m sprint; - pass basketball ball by both hands from the chest; - pass medicine ball (3 kg) by both hands from the chest; - bilateral handgrip; - 3-6-9 m drill test; - 30-seconds sprint test; - agility drill test; - 10x5 m sprint test	ICCs were ’very good’, the correlations were strong for each field test (r > 0.7). Only for the agility drill test the first repetition is statistically different compare to the second repetition (p = .015).	- 3 m sprint 1 [sec.] = 1.32 ± .18 - 3 m sprint 2 [sec.] = 1.32 ± .16; - 5 m sprint 1 [sec.] = 1.97 ± .26; - 5 m sprint 2 [sec.] = 1.95 ± .21; - 10 m sprint 1 [sec.] = 3.22 ± .27; - 10 m sprint 2 [sec.] = 3.20 ± .27; - 20 m sprint 1 [sec.] = 5.50 ± .42; - 20 m sprint 2 [sec.] = 5.50 ± .43; - pass the basketball ball to both hands from the chest 1 [m] = 11.24 ± 1.45; - pass the basketball ball between the hands from the chest 2 [m] = 11.18 ± 1.21; - pass the medicine ball both hands from the chest 1 [m] = 6.24 ± .50; - pass medicine ball both hands from the chest 2 [m] = 6.22 ± .59; - bilateral handgrip 1 [N] = 102.13 ± 26.68; - bilateral handgrip 2 [N] = 105.25 ± 26.10; - 30-seconds sprint test 1 [m] = 98.44 ± 6.36; - 30-second sprint test 2 [m] = 98.89 ± 6.00; - 10x5 m sprint test 1 [sec.] = 23.72 ± 1.67; - 10x5 m sprint test 2 [sec.] = 23.29 ± 1.48; - 3-6-9 m drill test 1 [sec.] = 15.81 ± 1.06; - 3-6-9 m drill test 2 [sec.] = 15.52 ± .71; - agility drill test 1 [sec.] = 28.61 ± 1.64; - agility drill test 2 [sec.] = 29.03 ± 1.68
**Ribeiro Neto et al. (2021) [9]**	To verify the relationships between the medicine ball throw (MBT) and wheelchair basketball mobility performance field tests and the shoulder and trunk peak torque in male and female beginner	37 wheelchair basketball athletes (18 female and 19 male). Functional classification: Female (class 1–2.5: 14, class 3–4.5: 4). Male (class 1–2.5: 14, class 3–4.5: 5). Disability, 4 myeomeningocele, 1 neurological disorder, 1 peripheral nerve injury, 1 poliomelitis and 30 spinal cord injury.	Tests applied was: -5m sprint, -5m sprint with ball, -20m sprint, -20m sprint with ball - Zig-zag agility, - Zig-zag agility with ball, - Medicine ballthrow.	The MBT presented significantly very high and perfect correlations with all wheelchair basketball field tests assessed (5-m sprint, 20-m sprint, and zig-zag agility test with and without a ball), and peak torque (R2 ranging from .810 to .995; P ≤ .05) for male and female athletes.	Field tests outcomes: -5m sprint(s): Female: 2.82(2.62–2.95) Male: 2.43(2.38–2.64) -5m sprint with ball(s): Female: 3.19(3.08–3.59) Male:2.97(.2.71–3.29) -20m sprint(s): Female: 7.40(.14–7.97) Male: 6.42(5.86–7.25) -20m sprint with ball(s) Female: 10.27(9.69–11.95) Male: 8.21(7.30–9.23) -Zig-zag agility(s) Female:20.66(20.09–21.95) Male: 18.81(18.25–21.41) -Ziga-zag with ball(s) Female:50.22(35.09–61.10) Male: 32.50(27.22–39.81) -Medicine ballthrow(m) Female:1.97(1.65–2.30) Male: 3.29(3.13–3.65)
**Weber et al.** **(2021) [26]**	to estimate the anaerobic power of wheelchair basketball athletes.	11 male wheelchair basketball athletes. Disability, 1 myeomeningocele, 1 muscle atrophy, 1 right lower limb amputation, 3 poliomelitis and 5 spinal cord injury. Functional classification(IWBF), 6 class 1, 2 class 1,5, 1 class 2,5, 1 class 3 and 1 class 4. Category A (1.0 to 2.5); Category B (3.0 to 4.5).	Field-based tests: -repeated sprints of 15 (S-15) and 20 (S-20) meters. -medicine ball chest pass test.	The time to complete the field tests (S-15 = 33 ± 5 s and S-20 = 42 ± 7 s), were higher than the 30 s WT, but the difference was statistically significant only for S-20. There was a strong correlation between performance in the medicine ball chest pass and PP in the WT.	Repeated sprints tests (s) -S-15: 32.7 (5.4) -S-20: 41.9 (7.3) Medicine ball pass(m): - 3.9(1.1)
**Soylu et al. (2021) [10]**	Investigate the relationship between athletic performance and physiological characteristics in wheelchair basketball (WB) athletes with different classification scores.	26 (24 male and 2 female) wheelchair basketball, athletes. The participants were divided into two functional categories: A (classes from 1.0 to 2.5; n = 13) and B (classes from 3.0 to 4.5; n = 13) according to the IWBF rules. The health conditions: spinal cord injury (n = 14), spina bifida (n = 3), lower limb amputations (n = 4), poliomyelitis (n = 2), cerebral palsy (n = 1) and other physical impairments (n = 2).	Testes applied: - 20-meter sprint test - Zone shot test -Shalom test	Category B athletes achieved significantly better results in the measurements of shoulder IR and ER muscle strength, aerobic and anaerobic capacity, and athletic performance while the grip strength was found similar in the categories. There was a significant correlation between the athletic performance and shoulder IR and ER muscle strength, and aerobic and anaerobic capacities in two categories. There was no significant relationship between grip strength and athletic performance parameters in two categories	- 20-meter sprint test(s) Category A: 7.47 (0.66) Category B: 6.03 (0.40) - Zone shot test(point): Category A: 17.15 (4.27) Category B: 27.38 (3.27) -Shalom test(s) Category A: 12.95 (1.15) Category B: 10.55 (0.73)
**Ali et al.** **(2021) [13]**	Identify some of the skills of running in a wheelchair and the accuracy of shooting skill from movement amongwheelchair basketball players,and the extent of the impact of special exercises in developing them	12 community as players of the Paralympic Subcommittee in Babil Province, wheelchair basketball, for the sports season 2019–2020.and the researchers chose his sample from the aforementioned society by (9) players, according to the medical classification.	Tests applied was: -Running in a wheelchair (15) of movement. -Running in a wheelchair (30 m) of stability. -Running in a wheelchair around a rectangle (50)m -Zigzag wheelchair run back and forth for a distance (20)m -Run windingly (10) m and return straight.	There is a positive effect of special exercises in developing all the skills of running on a wheelchair and shooting from the movement of players wheelchair basketball.	Field tests outcomes: -Running in a wheelchair (15) of movement(s): Before: 5.68(0.60) After: 5.29(0.55) -Running in a wheelchair (30 m) of stability(s): Before: 14.10(1.54) After: 13.54(1.34) -Running in a wheelchair around a rectangle (50)m Before: 33.55 (2.55) After: 35.39 2.20 -Zigzag wheelchair run back and forth for a distance (20)m Before: 25.28 (3.32) After: 27.84 (4.44) -Run windingly (10) m and return straight. Before: 23.45 (2.05) After: 22.11 (2.03) -shooting: Before: 8.66 (2.12) After: 10.33 (1.73)

PVA, paralyzed veterans of America; Class, sports classification, WB, wheelchair basketball, NWBA, National Wheelchair Basketball Association; IWFB, international wheelchair basketball federation; *FCS*, functional classification score; (d), dominant hand; (nd), nondominant hand; SS(1–3), category (A-B), functional categories, subscores; FM%, % fat mass; AnP, anaerobic performance; EG, experimental group; CG, control group; WMP, wheelchair mobility performance; MBT, medicine ball throw; S15 and S20, repeated sprints, TOT, total score (sum of three sub-scores), ICCs, interclass correlation coefficient; ES, effect size; s, seconds; m, meters; min, minimum; max, maximum; sec, seconds; mid, middle; n, numbers scores; N, newton; IR, internal rotation; ER, external rotation; WT, Wingate test; PP, peak power; MBT, medicine ball throw; Δ*t*, timing; f, push cycle frequency; Fp, progression force; sym, bilateral symmetry; CV, coeficients of variations.

The tests with the least number of applications were shooting test (free throw shooting, basket per minute, medicine ball throw, zone shot, spot shot, shot and lay-up) were presented in 14 studies [2,10,12–20,22,24,29]. Agility tests (slalom with and without ball, ball pick-up, 3-6-9 m drill test, agility drill test, agility T test, figure eight with ball test) were also used in 8 studies [2,9,10,12,16–18,22]. Drible and obstacle drible test presented in 5 studies [3,11,14,19,24]. Endurance test (6 min endurance race, suicide, yo yo 10 m recovery test, modified Cooper 12-minute test) were presented in 5 articles [2,12,17,18,22]. Lay-up tests in 2 articles [12,22]. Rebounding [14], bounce spot and bounce spin [19], catching [19], bilateral handgrip [16], envelope drill test [17], playing time [3] and wheelchair mobility performance WMP test [27] were presented by one article each. For more information about protocol tests, see Table 3.

**Table 3:** Skill tests protocol.

References	Test protocol
**Brasile. (1986) [14]**	- The obstacle dribble test was modified due to wheelchair use and also to comply with specific NWBA regulations regarding dribble execution. - Pass-for-accuracy test: was modified to give the Class I athlete a 5-foot throwing advantage. This was done because the original restraining line of 30 feet was deemed an impossible distance for these participants to score on a competitive basis with the Class III and Class II athletes. Thus, the Class I athlete threw from immediately behind a 25-foot restraining line, while the Class II and Class III athletes threw from behind the original 30-foot restraining line.
**Brasile. (1990) [24]**	- Pass for Accuracy* The subject must stay behind a restraining line 25 feet from the target. At a signal from the leader, the subject is to pass the basketball toward the target. Any form of the pass is acceptable (baseball, hook, or chest pass are suggested). The subject is allowed for 20 tosses. One trial of 10 passes with the dominant hand and one trial of 10 passes with the nondominant hand will be given. Each score will be recorded and judged accordingly. Either a hook or a baseball type throw is recommended. Subject may shoot a two-handed pass only for the dominant hand trial. - 20-Meter Dash The subject takes a position behind the out-of-bounds line at the end of the floor. Starts on the signal from the starter. Pushes the 20-meter distance as fast as possible. - Obstacle Dribble* Start at the right of the first obstacle. Subject maneuvers through the course as fast as possible pushing the wheelchair and dribbling the basketball, adhering to the NWBA rules for dribbling. At a signal from the leader, the subject pushes forward then pass to the left of the first wheelchair, then turns right and passes to the right of the next wheelchair, turns left and passes to the left of the next wheelchair, then turns right and circles the last wheelchair counterclockwise. The subject then pushes back to the starting line, staying outside of the wheelchairs. The subject pushes the course one more time. Each violation of the NWBA dribbling rule is an additional 5 seconds added to the individual’s time at the end of the test. Each time the individual, ball, or wheelchair touches an obstacle, an additional 1 second is added to the time at the end of the test. The subject may go after a loose ball and recover it. The subject must then return to the place where the error occurred and proceed along the course from that spot. The clock continues to run at all times. One trial is permitted. - Baskets per Minute The subject starts in a position facing the basket from behind the foul line. At a signal from the starter, the subject should proceed to shoot as many baskets as possible in 1 minute. Subjects may shoot from any point on the playing floor; all moves made during the test, including retrieval of a loose ball, must be in accordance with the NWBA rule for dribbling. There is a 1-point penalty for each violation of the dribbling rule. Score the total number of baskets made in the 1 minute allowed. The subject must retrieve his own rebounds under all circumstances. One trial of shots with the dominant hand and one trial of shots with the non-dominant hand will be given. Each score will be recorded and judged accordingly. - Spot Shot The subject starts at Spot 1. The subject shoots the basketball from Spot 1 and then does the same from Spots 2 through 7. The subject then works higher way back, starting at Spot 7. The subject receives 2 points for each basket made and 1 point for hitting the rim but not making the basket (if the ball hits the backboard and then the rim, it counts as 1 point). The subject receives 0 points for all other possibilities (e.g., hitting backboard but not rim, hitting net, missing basket completely).
**Vanlandewijck et al.(1999) [15]**	-20 m sprint test: The player takes a position behind the baseline. Following the signal of his partner, the player has to cover the 20 m distance as quickly as possible. Each player may have two attempts within the two minutes period. The best result is recorded. - Lay-up test: Two cones are positioned on the 3-point-line, perpendicular to the intersection of the sidelines of the free throw lane and the baseline. The player starts as indicated in Fig 1. The player has to make as many lay-ups as possible within two minutes. After each attempt, the player takes his own rebound, drives around the opposite cone with the ball, preparing for the next lay-up. The score is the total amount of attempts plus the total number of successful lay-ups. - Figure-eight + ball test: The starting position of the player is indicated in Fig 1. After the whistle of the trainer/coach, the player moves his wheelchair around the two cones in a figure of eight during one minute. The cones are positioned 5 m from each other, symmetrically to the centre line of the basketball court. The player has to control the ball while going around the cones. The score is the number of times the player can cover the 5 m distance. If, at the end whistle, the player crossed the centre line of the basketball court, this distance is valid. - Zone-shot test: The player starts at the foul line. Following the start signal, the player shoots as many baskets as possible from outside the free throw lane in two minutes. After each attempt the player has to shoot from a different, freely chosen zone. The score is the total amount of attempts plus the total number of successful throws in two minutes. - Figure-eight test: Except for the ball handling, this test is identical to test figure-eight + ball test. - Pass for accuracy test: A 30 cm square is marked on the wall of the sports hall. The centre of the square is at 1.2 m above the ground. Following the start signal of the trainer/coach, the player has to pass the basketball towards the target during two minutes. Any kind of pass is accepted with the restriction that the ball may not bounce before hitting the target. The player has to pass alternatively from behind the 4 and 8 m distance line. Every time the player hits the square from behind the 4 m line, one point is scored, and from behind the 8 m line two points are scored.
**Zwakhoven et al. (2003) [19]**	- Dribble. 1. The ball is dribbled in front and close to the side of the wheelchair; 2. The ball is played from the wrist with minimal elbow flexion; 3. Eyes are not fixed on the ball, player keeps overview of the game while dribbling; 4. The wheelchair is continuously positioned between the ball and a defensive player; 5. Player keeps on dribbling while moving the wheelchair. - Bounce stop. 1. The ball is dribbled once at the front axle of the wheelchair and caught with the same hand with full control of the ball; 2. The wheelchair is under control, after braking (stopping) using both hands; 3. The bounce-stop is done on the correct side of the wheelchair; 4. Eyes are not fixed on the ball, player keeps overview of the game - Bounce-spin. 1. The ball is dribbled once at the rear axle of the wheelchair and caught with the other hand with full control of the ball; 2. The wheelchair is spinned the amount of degrees necessary to execute the next movement; 3. The spin is done with a minimal displacement of the wheelchair while using both hands; 4. The bounce-spin can be done in combination with other movements; 5. Bounce-spin is done on the correct side of the wheelchair; 6. Eyes are not fixed on the ball; player keeps overview of the game.
**Doyle et al. (2004) [25]**	- 20m sprint: Participants were positioned at the starting line facing away from the finish line. They were asked to pivot their wheelchairs 180° and sprint as fast as possible over a 20-meter distance. The direction of the initial pivot was self-selected by the participants. The rear wheel axle was aligned with the start line of the 20-meter course. Participants positioned their bodies to be ready to react as quickly as possible to a siren that indicated the start of the test. The time between ’Get ready’ and the siren was randomized to eliminate preparatory strategies that would arise from being able to anticipate the siren. Electronic timing gates (Fitness Technologies, Adelaide, Australia) were placed to record times at 1-, 5- and 20-meter intervals. The timing gates interfaced with a personal computer running the Kinematic Measurement System (Innervations, Indiana, USA). Since this was a regular task performed by the players, there were no familiarization tests. Participants performed these trials using their own wheelchair of which some had a fifth rear wheel. Data were recorded for three sprints, and the best time was used for analysis.
**Harbalis et al. (2008) [11]**	- Dribbling Test. The dribbling test from international perspectives on adapted physical activity, a study of wheelchair basketball skills (Vanlerberghe & Slock, 1984) was used to assess dribbling performance. The test assesses the speed and dexterity of dribbling. It involves weaving in and around the cones as fast as possible, pushing the wheelchair, and dribbling the ball according to the ISMFF dribbling rules. The score for each participant is the time needed to complete the task. - Passing Test The passing test from the international perspectives on adapted physical activity, a study of wheelchair basketball skills (Vanlerberghe & Slock, 1984) was used to assess the passing performance. Participants placed their wheelchairs so that the front wheels were behind a line that was 2 m from the wall. On a verbal signal, the athlete passes the ball to the wall and catches it on the rebound in the air as many times as possible within a 15 seconds time frame. The score for each participant is the number of successful executions during this period.
**Skucas et al. (2009) [3]**	- Basic movements of the game: According to D. Byrnes and B. Hendrick (1994) methodology the following actions of the players (playmakers, forwards, centers) with different functions in the team were recorded in a special protocol: playing time, passing, dribbling, shooting and its efficiency, rebounding, and other important technical actions characterizing the activity of the players during the game. - Integral qualification, ability to play, and quality of play: were evaluated according to the methods of D. Byrnes and B. Hendrick (1994), giving high or low marks for each action: 1. Back picks + 4 2. 2-point field goals made +5 3. 2-point field goals missed—3 4. 3-point field goals made +6 5. 3-point field goals missed—4 6. Foul goals made +4 7. Foul goals missed –2 8. Offence rebounds +4 9. Defensive rebounds +4 10. Personal fouls—2 11. Assists +5 12. Turnovers –6 13. Bloked shots +5 14. Steals +5 15. Forced turnovers in defence +6 16. Technical fouls—10.
**Molik et al. (2010) [17]**	- 20 m sprint, two-handed chest pass (distance covered), slalom without the ball, slalom with the ball, modified Cooper test (line to line wheeling for a distance of 24 m each way), envelope drill (Ergun et al. 2008, Molik et al. 2008 [30], Vanlandewijck et al. 1999 [15], Ulatowski. 1963, Sobiecka. 1965, Vanlerberghe and Slock. 1987). The reliability for all of these tests has been documented in previous studies (Molik et al.2008 [30]). The envelope drill is another universal able-bodied basketball skill test and was adapted for wheelchair users. The test was adapted for wheelchair basketball from the traditional three loops to two loops but changing directions for the second loop. This adaptation requires the wheelchair athlete to have to turn their chair to the right and left–a potential discerning factor in classification.
**De Groot et al. (2012) [22]**	The sequence of tests was the same during trials one and two. Participants had standardized rest times between test items. Furthermore, the tire pressures in their chairs were checked and similar between trials. It took approximately 75 minutes to test five players with the complete test battery. - Pass-for-accuracy. Participants begin with the front wheels behind a line 7 m from the target. The participant has to pass the basketball 10 times towards the target. Any form of the pass is acceptable. Depending on where the ball hits the scoring board (Fig 1) participants score 3, 1 or 0. This test was previously described (Brasile, 1986 [14]; Brasile, 1990 [24]; Vanlandewijck et al., 1999 [15]) but without a clear scoring system. The tester records component scores and the end score is the sum of the scores of the 10 passes (range: 0–30). The domain tested is passing (accuracy). - 5-m sprint. The participant starts from a stationary position, with the front wheels behind the start line, and pushes 5 m as quickly as possible. Time is manually recorded with a stopwatch with a precision of 0.01 s and starts when the front wheels crossed the start line and stop when the front wheels cross the finish line. The test is performed three times, and the end score is the same as in the three trials. The tested domain is (start-up) speed. - Free-throw shooting. The participant shoots 10 free throws. To be able to discriminate between good, bad and very bad throws, the scoring is 3 –when the shot is a hit, 1 –when the ball touches the ring of the basket but is not a hit, 0 –when the ball does not touch the ring at all. The total score is calculated (range: 0–30), in which higher scores reflect a higher score. level of performance (Brasile, 1986 [14]). Shooting is the domain that is tested. - 20-m sprint with the ball. The participant starts with a ball from a stationary position and pushes 20 m as fast as possible, adhering to the IWBF rules for dribbling. Time is recorded by a stopwatch and starts when the front wheels cross the start line and stop when the front wheels cross the finish line. The end score is the time necessary to complete the 20 m. The tested domains are speed and ball handling. - Maximal pass. The participant begins in the middle of the baseline, front wheels behind the line, and has to pass a ball as far as possible from a stationary position. The first time the ball hits the floor should be between the lines of the basketball court. The distance between the participant and where the ball hits the floor is measured (in meters). The end score is the average distance of five passes. The domain that is tested is passing (explosiveness). - Slalom. From a stationary position, front wheels behind the start/finish line, the participant has to propel the wheelchair as fast as possible, first straightforwardly and thereafter through a slalom course of five cones, with a distance between the cones of 1.5 m, and back. The time to complete the test is recorded. The tested domains are speed and maneuverability. Lay-ups. The participant starts with the ball behind the 3-point line. The aim of this test is to score as many lay-ups as possible within a minute, adhering to the IWBF rules for dribbling. After each lay-up, the participant has to go back to the 3-point line and has to pick up the ball from a cone. Scoring: 3 – when the shot is a hit, 1 –when the ball touches the ring but is not hit, 0 –when the ball does not touch the ring at all (Vanlandewijck et al., 1999 [15]). Ball handling, shooting, and speed are the domains tested. - Pick up the ball. From a stationary position, the participant has to start propelling and must pick up four balls from the floor, twice with the left hand and twice with the right hand. After picking up the ball, the ball should be placed on the lap, and the participant has to push the wheelchair once before throwing the ball away. The total time to complete the test was recorded. The tested domains are ball handling and speed. - Spot shot. Five shots from four positions around the key, two at the top of the key (left and right), and two at the base of the key (left and right). Scoring: 3—when the shot is hit, 1—when the ball touches the ring but is not hit, 0 –when the ball does not touch the ring at all. The final score is the sum of the scores of the 20 shots (Brasile, 1986 [14]; de Groot et al., 2003 [29]; Vanlandewijck et al., 1999 [15]) (range: 0–60). Shooting is the tested domain. - Suicide. Maximal speed ladder-type test using all lines of the court. The player lines up on the baseline, on a start sign the player starts sprinting. First to the foul line and back, then to the half and back, then to the far foul line and back and the last lap is to the far baseline and back. The total time taken to complete the test is recorded. The domains tested are speed and endurance.
**Molik et al. (2018) [8]**	-Chest pass: Performed with the participant in their wheelchair with both feet placed on the footrest; the large wheel axle was lined up with the starting line; the participant was encouraged to use both arms equally to push the ball and the result was the maximum distance the ball travelled, best of three attempts. –Shooting: The participant took ten shots from five different places around the basket. The result was the number of successful baskets. – 5 m sprint: Performed with the large wheel axle lined up with the starting line; the participant pushed as hard and as fast as they could on a 5 m course and result was the time in seconds, fastest of two attempts. – 20 m sprint: With the large wheel axle lined up to the starting line, the participant pushed as hard and as fast as they could on a 20 m course; the result was the time in seconds, fastest of two attempts. –Slalom (with the ball): With the large wheel axle lined up with the starting line, the participant pushed as hard and as fast as they could on a slalom course while dribbling the ball; the result was the time in seconds, fastest of two attempts. –Slalom (without the ball): With the large wheel axle lined up with the starting line, the participant pushed as hard and as fast as they could on a slalom course; the result was the time in seconds, fastest of two attempts.
**Bergamini et al. (2015) [23]**	- 20 m Sprint Athletes started from a static position with the front wheels behind the start line and, after the start signal, pushed themselves for 20 metres as fast as possible. Time was manually recorded using a digital stopwatch (*t*20 mS). The test was performed twice and the trial corresponding to the shortest time of the athletes was also considered.
**Gil et al. (2015) [2]**	- The 20 m sprint with and without a ball In the sports hall, the basketball players performed a 20 m flat sprint test. In addition, they performed a similar test with a ball, adhering to the IWBF rules for dribbling (De Groot et al., 2012 [22]). The starting position of the players was 0.5 m before the first time light. All tests were performed three times with 2 min of recovery between. The best result of each test was used for further analysis. - T-test The participants began with the wheels 0.5 m from cone A, and completed the circuit as follows using the protocol by Sassi et al. (2009), modified to perform with a wheelchair and always using forward movements (Yanci et al., 2015 [28]). A-B displacement (9.14 m): At his/her own discretion, each subject moved quickly forward to cone B and touched the top with the right hand. B-C displacement (4.57 m): Facing forward, they moved to the left to cone D and touched the top with the left hand. C-D displacement (9.14 m): The participants then moved to the right to cone D and touched the top. D-B displacement (4.57 m): They moved back to the left to cone B and touched the top. B-A displacement (9.14 m): Finally, the participants moved as quickly as possible and returned to line A. All participants performed the test 3 times with at least 3 min rest between trials. The total distance covered was 36.56 m and the height of the cones was 0.3 m. Seven days later, the retest was performed under the same conditions. - Pick-up the ball From a stationary position the participant had to start propelling and pick up four basketball balls from the floor as previously described by De Groot et al. (2012) [22]. All participants performed the test 3 times with at least 3 min rest between trials. - Hand dynamometry test They squeezed the dynamometer (Jamar, USA) with a maximum isometric effort for 5 s with a rest period of at least 60 s and the highest value was used to determine maximal grip strength (kg). - Maximal pass As described by De Groot et al. (2012) [22], the participant began in the middle of the baseline, front wheels behind the line, and passed a basketball as far as possible from a stationary position. The distance between the participant and the spot where the ball hit the floor was measured (in meters). The final score was the average distance of five passes. - Medicine ball Using a similar position to the maximal pass, participants had to throw a 5 kg medicine ball as far as possible (Gonaus and Muller, 2012). The distance was measured in meters. Each participant made three attempts and the best was used for further analysis. - Yo-Yo intermittent recovery adapted test (Yo-yo ITa) The Level 1 version of the Yo-Yo test was completed according to previously described methods (Castagna et al., 2008). Due to the differences between running and propelling the wheelchair, the distance covered in the shuttle run was reduced to 10 m (Yanci et al., 2015 [28]). The total distance covered during the test was measured (meters). Heart rate (HR) was recorded at 5 s intervals by telemetry (Polar Team Sport System®, Polar Electro Oy, Finland) throughout the test. Before (pre-test) and immediately after the endurance test (post-test), earlobe capillary blood samples were obtained for the determination of lactate concentrations (Lactate Pro LT-1710®, ArkRay Inc Ltd, Kyoto, Japan).
**Yanci et al. (2015) [28]**	-Sprint Without and with ball: The subjects undertook a wheelchair sprint test consisting of three maximal sprints of 20 m, with a 120 s rest period between each sprint, enough time to return to the start and wait for their next turn. -Maximal sprint test with the ball: Performed using the same protocol and material. The participants started with a ball from a stationary position and pushed 20 m as fast as possible, adhering to the IWBF rules for dribbling. The test consisted of 3 maximal sprints with the ball over stretches of 20 m. -T-test: The participants began with the wheels 0.5 m from cone A, A-B displacement (9.14 m): At his/her own discretion, each subject moved quickly forward to cone B and touched the top with the right hand. B-C displacement (4.57 m): Facing forward they moved to the left to cone D and touched the top with the left hand. C-D displacement (9.14 m): The participants then moved to the right to cone D and touched the top. D-B displacement (4.57 m): They moved back to the left to cone B and touched the top. B-A displacement (9.14 m): Finally, the participants moved as quickly as possible and returned to line A. All participants performed the test 3 times with at least 3 min rest between trials. -Pick-up the ball: From a stationary position the participant had to start propelling and had to pick up four basketball balls from the floor, twice with the left hand and twice with the right hand. After picking up the ball, the ball had to be placed in the lap and the participant had to push the wheelchair once before throwing the ball. -Maximal pass: The participant began in the middle of the baseline, front wheels behind the line, and had to pass a basketball ball as far as possible from a stationary position. The distance between the participant and where the ball hit the floor was measured (in metres). The end score was the average distance of five passes. -Yo-Yo intermittent recovery test: Consisted of 10 m shuttle runs performed at increasing velocities with 10 s of active recovery between runs until exhaustion. Pushing speeds were dictated in the form of audio cues broadcast by a pre-programmed computer. The test was considered to have ended when the participant failed twice to reach the front line in time (objective evaluation) or felt unable to cover another shuttle at the dictated speed (subjective evaluation). The total distance covered during the test was measured.
**Cavedon et al.(2015) [20]**	All tests were performed according to de Groot et al. (2012) [22]. - 5m sprint: The player started from a stationary position, with the front wheels behind the start line and pushed for a distance of 5m as quickly as possible. The test was performed three times and the score was the average time of the three trials. -20m sprint with ball: The player started with a ball from a stationary position and pushed for a distance of 20m as fast as possible, adhering to the FIPIC rules for bouncing; the score was the time taken to complete the 20m. -Suicide: The player positioned himself on the baseline, pushing first to the foul line (free-throw line) and back, then to the half line and back, then to the far foul line (free-throw line) and back, then to the far baseline and back. The total time to complete the test was the score. -Maximal pass: The player sat stationary with the front wheels behind the baseline, attempting to throw the basketball ball as far as possible. The distance between the baseline and where the ball first hits the floor was measured. For this test three trials were performed, and the score was the average. -Pass for accuracy: Test the player, from behind a 4m distance line, had to pass the basketball 10 times towards a 30cm square target (with a 2cm border) marked on the wall of the sports hall. The centre of the square was at 1.2m above the ground. Any form of pass was acceptable with the restriction that the ball may not bounce before hitting the target. Players scored 3, 1 or 0 points when they hit the target, the target border, or no target, respectively. The score was the sum of the points of the 10 passes (range: 0–30). -Spot shot: The player had to perform five shots from four positions around the lane (i.e. the area between the free-throw line and the base line), two at the top of the lane (left and right) and two at the base of the lane (left and right). -lay-ups: The players started with the basketball behind the 3-point line aiming to score as many lay-ups as possible within a minute. After each lay-up participants were asked to go back to the 3-point line and to pick up the ball from a cone.
**de Witte et al. (2017) [27]**	- WMP test; The WMP test for wheelchair basketball consisted of 15 activities with a standardized rest period between the activities. The WMP test is divided into four main groups. Group (1): Separate activities containing a 12 m sprint, a rotation with a curve (circumference) of 12 m (clockwise/ counterclockwise) and a turn on the spot (clockwise/counterclockwise); Group (2): Combined activities containing the same activities as group 1, combined with starts and stops in between; Group (3): Specific skills consisting of a tik-tak box, which means performance of short movements forward and backward alternated with collisions against a stationary object. Group (4): A 12 m sprint and rotation (clockwise/counterclockwise) with a curve (circumference) of 12 m performed with ball possession (dribble).
**Cavedon et al.(2018) [21]**	All tests were performed according to De Groot et al. (2012) [22].
**Yüksel & Sevindi. (2018) [12]**	-Pass for distance: The subject places the wheelchair so that the front wheels are behind the base line. Using the chest pass, he tries to pass the ball as far as possible. Subject was performed six attempts and the total of the measured distance was recorded. -20-m speed: The player takes a position behind the baseline. Following the signal of his partner, the player has to cover the 20 m distance as quickly as possible. Each player may have two attempts within the two minute period. The best result is recorded. -Slalom without the ball and slalom with the ball: The reliability for all of these tests has been documented in previous studies Molik et al. (2010) [17]. -Lay up: Two cones are positioned on the 3-point line, perpendicular to the intersection, of the side lines of the free throw lane and the baseline. The subject takes position out of the 3-point line and starts with the signal to make as many lay-ups as possible within two minutes. After each attempt, he takes his own rebound, dribbles the ball around the opposite cone, preparing for the next lay-up. The score was the total amount of the attempts, plus the total number of the successful lay ups. -Zone shot: The player starts at the foul line. Following the start signal, the player shoots as many baskets as possible from outside the free throw lane in two minutes. After each attempt the player has to shoot from a different, freely chosen zone. The score is the total amount of attempts plus the total number of successful throws in two minutes. -Pass for accuracy: Pass for accuracy test were recorded according to the methodology of Ergun et al. (2008).
**Tachibana et al. (2019) [18]**	- 20 m sprint The participant started from a stationary position behind the starting line and, following a signal, sprinted a straight 20 m distance, as fast as possible. The sprint was performed twice within 2 min, and the best result was recorded. - Agility T-Test Protocol by Yanci et al (2015) [28]. The participants stood still, with the caster wheels behind the starting line, facing pylon A. After a signal, the participant pushed forward as quickly as possible to pylon B and touched the top of it. The player then turned left and moved to pylons C, B, and D (in order), touching the top of each pylon, and finally returned to pylon A. The time to complete the sequence was measured. The participant performed the test twice, and the best result was recorded. - Figure-Eight with a Ball Test Protocol by Vanlandewijck et al (1999) [15]. Following a signal, the participant moved the wheelchair around two cones in a figure-eight shape while dribbling. The participant was required to push as fast as possible, adhering to the IWBF rules for dribbling. The cones were positioned 5 m apart from each other. The time taken to complete five laps was determined. The participant performed the test twice, and the best result was recorded. - Yo-Yo 10 m Recovery Test Protocol by Yanci et al (2015) [28]. As the propelling speed is slower than the running speed, the distance covered in the shuttle run was reduced to 10 m (it was 20 m in the original version). The participant was required to complete as many shuttle runs as possible, going back and forth between the lines in accordance with a beeping sound. The test ended when the participant failed twice to reach the goal line in time. The total distance covered during the test was recorded. The total distance covered during the test was recorded. - Maximal Pass The participant positioned the front casters behind the baseline and threw a ball as far as possible from a stationary position. We evaluated three types of passes: chest, baseball, and hook pass. The chest pass originated from the chest and used both hands. The baseball pass was a one-handed pass that used the same motion as that of the baseball throw. Hook pass was also a one-handed pass, starting from the lateral side of the player’s trunk. The participants held the ball at shoulder level with one arm extended, then lifted the arm and passed the ball over their head, followed by a throw to the target. The participants performed each of the three passes twice. The distance between the baseline and the point at which the ball hit the floor was measured.
**Marszałek et al. 2019 [16]**	All procedures and descriptions of field-based tests were explained and checked in terms of validity by Marszałek et al. (2019) [16]: - 3 m sprint, 5 m sprint, 10 m sprint, 20 m sprint tests The participants pushed as hard and as fast as they could over the 3, 5, 10 or 20 m course. The result was the time in seconds (the fastest of the two attempts). - Bilateral handgrip Participants squeezed a manual hand grip dynamometer DR3 with the WTP003 tensometer using software version 3.1. They performed the test sitting in their wheelchairs with the arm tested fully extended and without touching the wheelchair. The result was the combination of the value for the right and left hand. - Basketball chest pass test and medicine ball (3 kg) chest pass test Participants were in their wheelchairs with their feet placed on the footrest. The rear wheel axle was aligned with the starting line. Participants were encouraged to perform the task using arms as symmetrically as possible. The result was the distance covered by the ball, the best out of three attempts, measured with a tape from the starting line to the place where the ball fell. The measurement error was ±5 cm. - 30-s sprint test The participants propelled their wheelchairs as fast as they could over a distance of 20 m, turned and returned for 30 s. The result was the distance achieved in meters. There was only one attempt. - Agility drill test The participants propelled as fast as they could over the 12-m course in a straight line, came back to start a slalom (four cons) and returned through the slalom. Then, they went straight over the 12-m course and came back. The result was the time in seconds (the fastest of the two attempts). - 3-6-9 m drill test The participants propelled as fast as they could over the 3-m course and came back to the starting line, then covered the distance of 6 m and came back to the starting line. Finally, they propelled for 9 m and returned to the starting line. The result was the time in seconds (the fastest of the two attempts). - 10 × 5 m sprint test The participants propelled as fast as they could 10 times over the 5-m course. The result was the time in seconds (the fastest of the two attempts).
**Ribeiro Neto et al.(2021) [9]**	-5m sprint and the 20-m sprint: tests with and without the ball were performed. Participants started from a stationary position, with the front wheels behind the starting line, pushing as rapidly as possible for 5 and 20 m, respectively. -Zig-Zag Agility Test: Participants started from a stationary position, with front wheels behind the start line, pushed the wheelchair as fast as possible while completing a preestablished circuit. The zig-zag agility test was performed with and without the ball. - MBT Testing: The participant had to throw a 5-kg medicine ball with a 2-arm overhand as far as possible from a stationary position, with one of the researchers holding the wheelchair in place.4,5 The distance between the participant and the spot where the ball hit the floor was measured (in meters). Each participant made 3 attempts, interspersed by 2-minute rest intervals. The longest distance was retained for further analysis.
**Weber et al.** **(2021) [26]**	-Sprint 15m and 20m: The sprints protocols were adapted from the RAST test (6 sprints with 10 seconds intervals) (Draper & Whyte, 1997). Two cones were positioned at the end of a 15 and a 20 meters long horizontal line. Other two cones were placed two meters after the end to prevent athletes from slowing down before the measuring distance
**Soylu et al. (2021) [10]**	- 20-meter sprint test: The best result of this test was used for further analysis (Molik et al., 2010 [17]; Vanlandewijck et al., 1999 [15]). - Zone shot test: Test was conducted to evaluate the shooting skills of the athletes. The athletes were asked to take the start position behind the foulline. With the “start” command, the athletes were asked to shoot for 2 min as many shoots as possible from outside the free throw line, and to rebound their shoots each time. - Slalom test: Test was conducted in order to measure the wheelchair riding skills of the athletes. Five cones were placed on the field, the first one of which was 1.5 m away from the baseline, with a 1.5 m distance between each. The athletes were asked to move forward among these cones with slaloms, and to finish the field after turning back behind the last cone and moving back among the cones with slaloms and passing the baseline.
**Ali et al.** **(2021) [13]**	All tests were performed according to Zafir H.O.(2007) -Running in a wheelchair (15) of movement: -Running in a wheelchair (30 m) of stability: -Running in a wheelchair around a rectangle (50)m: -Zigzag wheelchair run back and forth for a distance (20)m: -Run windingly (10) m and return straight: - Shooting for accuracy: used the shooting from the movementtest Ahmed A.M.(2013).

### Results of the skill tests

Table 2 shows the difference in test results between the levels of the sport class, so that the highest classes showed better performance results [2,10,11,14,16–18,24]. Differences are also observed when considering the level of training, high-level athletes showed better results when compared to amateur athletes [12,19]. Bergamini et al (2015) [23] assessed strength and power between different injury levels and observed higher levels of strength and power as a result (Δ*t*: push cycle duration; *f*: push cycle frequency; *Fp*: peak progression force; *sym*: bilateral symmetry index) better performance in propulsion tests on athletes who are introduced to the training group. One of the articles found no significant difference between the elite and sub-elite groups (classes IWBF and NWBA) in all subscores and scores [19]. In another study it was observed that there is a difference between the performance in different positions of the players in the passing and dribbling tests (p <0.05), in which central attackers showed better results compared to the other positions [3]. The study by Ribeiro Neto et al (2021) [9] found a difference in strength and power performance between the gender and a significant correlation of the medicine ball test with all other wheelchair basketball field tests evaluated (R2 ranging from 0.810 to 0.995; P ≤ 0.05).

### Skills assessed

Table 4 shows the skills explored in each study in its battery of tests. Pass skill has the highest prevalence in studies [2,3,12,14–19,22,24] followed by speed, agility, dribbling, and shooting [2,10,12–14,16–18,23]. Force [2,9,16] is the least explored skill followed by Pick-up [2,21,22,28] in testing during studies.

**Table 4:** Ability of wheelchair skills tests.

References	agility	passing	pick-up	shooting	speed	force	dribbling
**Brasile (1986) [14]**		xx		x			x
**Brasile (1990) [24]**		x		x			x
**Vanlandewijck et al. (1999) [15]**	x	x		x	x		
**Zwakhoven et al. (2003) [19]**	x	x	x	x			x
**Doyle et al. (2004) [25]**					x		
**Harbalis et al. (2008) [11]**		x					x
**Skucas et al. (2009) [3]**		x		x	x		x
**Molik et al. (2010) [17]**	x	x			x		
**De Groot et al. (2012) [22]**	x	x	x	x	x		
**Bergamini et al. (2015) [23]**					x		
**Gil et al. (2015) [2]**	x	x	x		x	x	
**Yanci et al. (2015) [28]**	x	x	x		x		
**Cavedon et al. (2015) [20]**		x		x	x		
**de Witte et al. (2017) [27]**	x				x		
**Yüksel and Sevindi. (2018) [12]**	x	x		x	x		
**Cavedon et al. (2018) [21]**	x	x	x	x	x		
**Molik (2018) [8]**	x	x		x	x		
**Tachibana et al. (2019) [18]**	x	x			x		
**Marszałek et al. (2019) [16]**	x	x			x	x	
**Ribeiro Neto et al.(2021) [9]**	x				x	x	
**Weber et al. (2021) [26]**					x		
**Soylu et al. (2021) [10]**	x			x	x		
**Ali et al. (2021) [13]**	x			x	x		

### Quality assessment

Through subjective evaluation of the methodological quality of studies using the AXIS tool, we identified that, in general, the articles included in this systematic review have a low risk of methodological bias. Assessing methodological quality of studies is important because results that come from studies with low risk of bias are more reliable, and for a systematic review, this point is important because it makes the scientific evidence stronger.

## Discussion

The aim of this systematic review was to identify, describe and synthesize the skill tests utilized in wheelchair basketball. To the best of our knowledge, no study was found with similar objectives. Therefore, our main findings are: a) the most explored skills were agility, pass and speed and the most frequent test was the pass tests and sprint tests, b) Associations were found between the sports classes and performance in field tests, c) The most used tests for each skill were: pass accuracy and maximum pass, 20m sprint test with and without the ball, slalom test, obstacle dribbling tests and free throw and spot shot.

### Skill tests: Main tests and skills assessed

Pass and sprint tests were the most frequently cited in the articles included here. The pass is a skill required throughout all wheelchair basketball games [31]. During the completion of the pass, different muscle groups are involved and trunk muscles are very important to stabilize the body; consequently, athletes of different classes will be presented with different ways of performing the pass and different performances [32]. Thereby, assessing the pass is important to improve the training of this skill and improve individual and collective performance. Pass tests assess the athlete’s ability to perform these activities and can also be used as a way of assessing upper limb function [18]. In a study, the maximal passes test of one hand (baseball and hook passes) was used and it was possible to identify differences between classification levels [18], which allows us to say that through this evaluation we can have an answer as to the degree of functionality of the upper limbs of wheelchair basketball athletes.

There are several passing techniques, which will be used according to the game situation [33], and in the case of wheelchair basketball, it will also vary according to the sport class. So, which test to use? The answer to this question can be found in the study by Izzo and Russo (2011) who evaluated passing techniques in 150 games in the high-level Italian league, NCAA and NBA. They found that the two-handed chest pass was the most common and easiest to perform during matches [34]. These results indicate that, taking into account that the two-handed chest pass is the most performed in matches, the two-handed chest pass test would be the most indicated to evaluate this skill. However, it is noteworthy that in wheelchair basketball this result may not be the same, since during the pass the athlete may be touching the chair, for example, and no study has observed which passing technique is the most used in games of Wheelchair basketball.

Sprint tests measure speed to cover a predetermined distance, cardiovascular capabilities, and performance. Yanci et al. (2015) [28], used T-test and Yo-Yo 10 m recovery test to evaluate agility and endurance performance of wheelchair basketball athletes and identified that the tests are reliable and therefore can be appropriate instruments for measuring physical fitness in wheelchair basketball [28]. The Yo-Yo test assesses an individual’s ability to perform intense exercise intermittently. Consequently, leading to the end of the test a predominance of the aerobic metabolic pathway [35]. In the version described by Yanci (2015) [28], due to the difference between running and wheel propulsion, the fixed distance to be covered during the test was reduced to 10 meters.

In wheelchair basketball there is an intermittent feature, where athletes alternate short and intense efforts, acceleration, deceleration, change of direction with active or passive recovery periods, with expressive participation of aerobic capacity during the match [28,36]. The training and play of wheelchair basketball are based on the aerobic pathway to provide athletes with energy [37]. Furthermore, the development of such a pathway can contribute to recovery between intermittent efforts and, consequently, to maintaining a higher intensity for a longer period of time, contributing to better endurance performance [38,39].

Evaluation of endurance performance becomes difficult in terms of the different methodologies applied, with different tests and variables analyzed (VO2, lactate, distance, heart rate) [28,36,37,40]. Hutzler (1993), comparing the aerobic and anaerobic performance of 11 elite Israeli wheelchair basketball players using an arm ergometer. Ergometric tests included a continuous aerobic maximum work capacity test and a 30 second all-out anaerobic test in the arm. The author concludes that the result of the arm ergometer may have a limited performance value for athletes.

To assess the aerobic capacity of the players, a suicide test was performed in two studies [20–22]. As well as, Yanci (2015) [28] and Tachibana (2019) [18] to analyze the physical characteristics measured by sprint, agility, strength and endurance with field tests on wheelchair basketball players demonstrated that the tests are reliable and can be appropriate instruments for coaches and athletes measuring endurance performance in WB. Yüksel and Sevindi. (2018) [12] used the 6-minute endurance running test to assess athletes’ endurance performance.

Sprinting is another important skill in wheelchair basketball, throughout the game athletes need to be at speed and perform other activities at the same time as they carry out wheelchair propulsion, and this skill is closely linked to performance [41]. Taking into account this characteristic, the interesting thing for this skill is to use tests that assess speed at the same time as other activities are performed, to simulate what could happen in a match. In this sense, the maximum speed test of 20 meters with the ball is interesting for this skill and also the WMP test because it is a battery of tests that simulate different game situations while the athletes perform sprints.

Both passing ability and speed are fundamental skills in wheelchair basketball and evaluating them provides an overview of an athlete’s performance on the court. The protocols for all tests are described in Table 3.

### Sport class, training level, and skill tests

Based on the results presented in this review, low-sport-class athletes (less functionality) showed worse results compared to high-class athletes (more functionality). These results are particularly interesting when we talk about evidence-based classification, which seeks to classify athletes based on empirical evidence [42]. In this sense, further investigation is need about skill tests as tools during the classification process because most are not time-consuming and practical. In fact some studies have already suggested the potential of using skill tests in the classification of wheelchair basketball athletes and have shown positive results [27,43,44].

For this to be a reality, it is still necessary to delimit parameters that define each sport class, with well-defined values (minimum and maximum value). Thus, the ideal for the use of these tests of skill during the classification would be the creation of a battery of tests with specific and well-defined objectives and the creation of tables of values, delimiting and characterizing each class based on the parameters to be adopted.

A similar phenomenon occurs when observing the level of training of athletes, those who have a higher level of training showed better results in skills tests. This result was already expected as the skills can be improved through training. Still, this result is interesting for structuring the training. Based on the results of skill tests, coaches can meet the specific demands of athletes, the tests themselves can be used as training programs.

### Limitations of the review

As it is a systematic review, our limitations are related to the studies that are inserted here. In addition, even though the sample is composed of a specific population (wheelchair basketball athletes) it is heterogeneous regarding training level, sport class, age, which may have affected the general interpretation.

### Implications for research and practice

Our study describes and synthesizes the main skills tests found in the literature. The links between results of skill tests and sport classification / training level made in this work, provide theoretical reference for future research in this field of study (both in sports classification and training). Currently, studies have used skill tests and collected results beyond what is generally found in the general literature (results are often described through the time the test is performed and the distance covered). Through the aid of technology (such as the use of accelerometers), these studies have analyzed linear acceleration and angular velocity [27,43–46].

With the results found here, with further investigation the skills tests could be considered for use during the evidence-based classification process of wheelchair basketball. In addition, these tests can be used by trainers to assess the skills that need to be improved in training, thus developing specific training circuits for each demand.

## Conclusions

This review presents the different field tests used in wheelchair basketball assessments. The most evaluated skills were speed through the 20m speed test, agility through the Slalon test, passing through the pass for accuracy test and throwing through the free throw shooting test. The use of specific skill tests facilitates the creation of reference standards and possible comparisons of athletes and, thus, enables better training conditions with the aim of meeting the specific demands of each athlete and team.

## Supporting information

S1 Table Appraisal tool for Cross-Sectional Studies (AXIS). (DOCX)

S2 Table PRISMA 2009 checklist. (PDF)
