# Supplementary material for: Wheelchair skill tests in wheelchair Basketball: A systematic review
Source: PLoS One. 2022 Dec 1;17(12):e0276946. doi: 10.1371/journal.pone.0276946 (PMC9714714; doi:10.1371/journal.pone.0276946)
Supplement: S1 Table — (DOCX) [file pone.0276946.s001.docx]

**Supporting information 1**. Appraisal tool for Cross-Sectional Studies (AXIS)

| **Question** | Basile (1986) | Basile (1990) | Zwakhoven et al. (2003) | Doyle et al. (2004) | Harbalis et al. (2008) | Yüksel and Sevindi. (2018) | Skucas et al. (2009) | Molik et al. (2010) | De Groot et al. (2013) | Bergamini et al. (2015) | de Witte et al. (2017) | Tachibana et al. (2019) | Gil et al. (2015) | Marszałek et al. (2019) |
| --- | --- | --- | --- | --- | --- | --- | --- | --- | --- | --- | --- | --- | --- | --- |
| **Introduction** |  |  |  |  |  |  |  |  |  |  |  |  |  |  |
| 1. Were the aims/objectives of the study Y clear? | | Y | Y | Y | Y | Y | Y | Y | Y | Y | Y | Y | Y | Y |
| **Methods** | |  |  |  |  |  |  |  |  |  |  |  |  |  |
| 2. Was the study design appropriate for the Y  stated aim(s)? | | Y | Y | Y | Y | Y | Y | Y | Y | Y | Y | Y | Y | Y |
| 3. Was the sample size justified? N | | N | N | N | N | Y | N | Y | N | N | N | Y | N | Y |
| 4. Was the target/reference population | |  |  |  |  |  |  |  |  |  |  |  |  |  |
| clearly defined? (Is it clear who the research Y | | Y | Y | Y | Y | Y | Y | Y | Y | Y | Y | Y | Y | Y |
| was about?) | |  |  |  |  |  |  |  |  |  |  |  |  |  |
| 5. Was the sample frame taken from an | |  |  |  |  |  |  |  |  |  |  |  |  |  |
| appropriate population base so that it closely Y  represented the target/reference population | | Y | Y | Y | Y | Y | Y | Y | Y | Y | Y | Y | Y | Y |
| under investigation? | |  |  |  |  |  |  |  |  |  |  |  |  |  |
| 6. Was the selection process likely to select | |  |  |  |  |  |  |  |  |  |  |  |  |  |
| subjects/participants that were representative Y  of the target/reference population under | | Y | Y | Y | Y | Y | Y | Y | Y | Y | Y | Y | Y | Y |
| investigation? | |  |  |  |  |  |  |  |  |  |  |  |  |  |
| 7. Were measures undertaken to address and N | | N | N | N | N | N | N | N | N | N | N | N | N | N |
| categorise non-responders? R | | R | R | R | R | R | R | R | R | R | R | R | R | R |
| 8. Were the risk factor and outcome variables | |  |  |  |  |  |  |  |  |  |  |  |  |  |
| measured appropriate to the aims of the Y | | Y | Y | Y | Y | Y | Y | Y | Y | Y | Y | Y | Y | Y |
| study? | |  |  |  |  |  |  |  |  |  |  |  |  |  |
| 9. Were the risk factor and outcome variables | |  |  |  |  |  |  |  |  |  |  |  |  |  |
| measured correctly using instruments/ Y  measurements that had been trialled, piloted | | Y | Y | Y | Y | Y | Y | Y | Y | Y | Y | Y | Y | Y |
| or published previously? | |  |  |  |  |  |  |  |  |  |  |  |  |  |
| 10. Is it clear what was used to determined | |  |  |  |  |  |  |  |  |  |  |  |  |  |
| statistical significance and/or precision Y | | Y | Y | Y | Y | Y | Y | Y | Y | Y | Y | Y | Y | Y |
| estimates? (eg, p values, CIs) | |  |  |  |  |  |  |  |  |  |  |  |  |  |
| 11. Were the methods (including statistical | |  |  |  |  |  |  |  |  |  |  |  |  |  |
| methods) sufficiently described to enable Y | | Y | Y | Y | Y | Y | Y | Y | Y | Y | Y | Y | Y | Y |
| them to be repeated?  **Results** | |  |  |  |  |  |  |  |  |  |  |  |  |  |
| 12. Were the basic data adequately  described? | Y | Y | Y | Y | Y | Y | Y | Y | Y | Y | Y | Y | Y | Y |
| 13. Does the response rate raise concerns  about non-response bias? | N | N | N | N | N | N | N | N | N | N | N | N | N | N |
| 14. If appropriate, was information about | N | N | N | N | N | N | N | N | N | N | N | N | N | N |
| non-responders described? | R | R | R | R | R | R | R | R | R | R | R | R | R | R |
| 15. Were the results internally consistent? | Y | Y | Y | Y | Y | Y | Y | Y | Y | Y | Y | Y | Y | Y |
| 16. Were the results for the analyses Y  described in the methods, presented? | | Y | Y | Y | Y | Y | Y | Y | Y | Y | Y | Y | Y | Y |
| **Discussion** | |  |  |  |  |  |  |  |  |  |  |  |  |  |
| 17. Were the authors’ discussions and Y conclusions justified by the results? | | Y | Y | Y | Y | Y | Y | Y | Y | Y | Y | Y | Y | Y |
| 18. Were the limitations of the study Y | | N | N | Y | Y | N | N | N | Y | Y | Y | Y | Y | Y |
| discussed?  **Other** | | | | | | | | | | | | | | |

1. Were there any funding sources or conflicts of interest that may affect the authors’ interpretation of the results?

N N N N N N N N N N N N N N

1. Was ethical approval or consent of participants attained?

N N N N N R R R R R

N N

Y R R

Y Y Y Y Y Y

Caption: Y = Yes, N = No and NR = Not Reported.

**Supporting information 1(continuation)**. Appraisal tool for Cross-Sectional Studies (AXIS)

| **Question** | Vanlandewijck (1999) | Yanci (2015) | Cavedon (2015) | Cavedon (2018) | Yüksel and Sevindi. (2018) | Molik et al. (2013) | Ribeiro Neto (2021) | Weber (2021) | Soylu et al. (2021) | Ali (2021) |
| --- | --- | --- | --- | --- | --- | --- | --- | --- | --- | --- |
| **Introduction** |  |  |  |  |  |  |  |  |  |  |
| 1. Were the aims/objectives of the study Y clear? | | Y | Y | Y | Y | Y | Y | Y | Y | Y |
| **Methods** | |  |  |  |  |  |  |  |  |  |
| 2. Was the study design appropriate for the Y  stated aim(s)? | | Y | Y | Y | Y | Y | Y | Y | Y | Y |
| 3. Was the sample size justified? N | | N | N | N | N | Y | N | Y | N | N |
| 4. Was the target/reference population | |  |  |  |  |  |  |  |  |  |
| clearly defined? (Is it clear who the research Y | | Y | Y | Y | Y | Y | Y | Y | Y | Y |
| was about?) | |  |  |  |  |  |  |  |  |  |
| 5. Was the sample frame taken from an | |  |  |  |  |  |  |  |  |  |
| appropriate population base so that it closely Y  represented the target/reference population | | Y | Y | Y | Y | Y | Y | Y | Y | Y |
| under investigation? | |  |  |  |  |  |  |  |  |  |
| 6. Was the selection process likely to select | |  |  |  |  |  |  |  |  |  |
| subjects/participants that were representative Y  of the target/reference population under | | Y | Y | Y | Y | Y | Y | Y | Y | Y |
| investigation? | |  |  |  |  |  |  |  |  |  |
| 7. Were measures undertaken to address and N | | N | N | N | N | N | N | N | N | N |
| categorise non-responders? R | | R | R | R | R | R | R | R | R | R |
| 8. Were the risk factor and outcome variables | |  |  |  |  |  |  |  |  |  |
| measured appropriate to the aims of the Y | | Y | Y | Y | Y | Y | Y | Y | Y | Y |
| study? | |  |  |  |  |  |  |  |  |  |
| 9. Were the risk factor and outcome variables | |  |  |  |  |  |  |  |  |  |
| measured correctly using instruments/ Y  measurements that had been trialled, piloted | | Y | Y | Y | Y | Y | Y | Y | Y | Y |
| or published previously? | |  |  |  |  |  |  |  |  |  |
| 10. Is it clear what was used to determined | |  |  |  |  |  |  |  |  |  |
| statistical significance and/or precision Y | | Y | Y | Y | Y | Y | Y | Y | Y | Y |
| estimates? (eg, p values, CIs) | |  |  |  |  |  |  |  |  |  |
| 11. Were the methods (including statistical | |  |  |  |  |  |  |  |  |  |
| methods) sufficiently described to enable Y | | Y | Y | Y | Y | Y | Y | Y | Y | Y |
| them to be repeated? | |  |  |  |  |  |  |  |  |  |
| **Results** | |  |  |  |  |  |  |  |  |  |
| 12. Were the basic data adequately  described? | Y | Y | Y | Y | Y | Y | Y | Y | Y | Y |
| 13. Does the response rate raise concerns  about non-response bias? | N | N | N | N | N | N | N | N | N | N |
| 14. If appropriate, was information about | N | N | N | N | N | N | N | N | N | N |
| non-responders described? | R | R | R | R | R | R | R | R | R | R |
| 15. Were the results internally consistent? | Y | Y | Y | Y | Y | Y | Y | Y | Y | Y |
| 16. Were the results for the analyses Y  described in the methods, presented? | | Y | Y | Y | Y | Y | Y | Y | Y | Y |
| **Discussion** | |  |  |  |  |  |  |  |  |  |
| 17. Were the authors’ discussions and Y conclusions justified by the results? | | Y | Y | Y | Y | Y | Y | Y | Y | Y |
| 18. Were the limitations of the study Y | | N | N | Y | Y | N | N | N | Y | Y |
